# Investigating repetitive transcranial magnetic stimulation–induced interhemispheric changes in stroke: a transcranial magnetic stimulation and fNIRS study

**DOI:** 10.1117/1.NPh.13.S1.S13010

**Published:** 2026-02-10

**Authors:** Siqi Yang, Yaomei Li, Fen Zhang, Geert Verheyden, Zhi Chen, Yongfang Li, Yawen Yang, Yang Liu, Chuanxin M. Niu, Jixian Wang, Qing Xie

**Affiliations:** a Ruijin Hospital, Shanghai Jiao Tong University School of Medicine, Department of Rehabilitation Medicine, Shanghai, China; b Shanghai Ruijin Rehabilitation Hospital, Department of Rehabilitation Medicine, Shanghai, China; c KU Leuven, Department of Rehabilitation Sciences, Leuven, Belgium; d KU Leuven, LIBIS, Leuven, Belgium

**Keywords:** interhemispheric inhibition, functional connectivity, stroke, transcranial magnetic stimulation, functional near-infrared spectroscopy

## Abstract

**Significance:**

Interhemispheric imbalance after stroke impedes motor recovery. Although repetitive transcranial magnetic stimulation (rTMS) shows therapeutic potential, the neural mechanisms underlying its effects—particularly on cortical excitability and interhemispheric interaction—remain unclear. Understanding these mechanisms is essential for optimizing personalized neuromodulation strategies in stroke rehabilitation.

**Aim:**

To explore how low-frequency rTMS facilitates motor recovery post-stroke, especially in regulating interhemispheric inhibition (IHI), functional connectivity, and cortical excitability, we used a multimodal approach that included paired-pulse TMS, functional near-infrared spectroscopy (fNIRS), and motor outcomes.

**Approach:**

A total of 27 patients with stroke were randomized to receive 2 weeks of either real (n=14) or sham (n=13) 1 Hz rTMS over the contralesional primary motor cortex (M1). Paired-pulse TMS was used to measure IHI and laterality quotient (LQ), whereas fNIRS was used to measure resting-state functional connectivity (RSFC). Clinical scales were also used to assess behavioral-level motor functioning.

**Results:**

Compared with the sham group, rTMS significantly modulated IHI, as evidenced by a reduction in inhibitory drive from the stimulated contralesional M1 to ipsilesional M1 (mean difference = 15.57%, p=0.048). In parallel, RSFC analysis revealed decreased connectivity from contralesional M1 to ipsilesional premotor and supplementary motor areas [F(2,50)=7.704, p=0.006, false discovery rate–corrected]. Between-group comparisons further showed greater LQ improvements in the rTMS group than in the sham group. Changes in selected neurophysiological measures were significantly correlated with improvements in upper limb motor function.

**Conclusions:**

We showed that low-frequency rTMS promotes motor recovery after stroke by rebalancing cortical excitability and reducing maladaptive connectivity patterns. The integration of TMS and fNIRS provides converging evidence for rTMS-induced cortical plasticity and highlights the potential of these tools for guiding personalized neuromodulation strategies in stroke rehabilitation.

## 1 Introduction

Stroke stands as the foremost cause of disability among adults worldwide.^1^ Despite undergoing standard rehabilitation therapy, fewer than 15% of stroke survivors achieve complete restoration of motor function on the upper limb.^2^ Given the essential role of upper limb involvement in daily activities, restoring it has persistently been a central and challenging goal in post-stroke rehabilitation.^3^ To extend the therapeutic horizons of conventional rehabilitation, repetitive transcranial magnetic stimulation (rTMS) has gained increasing attention in the field of neurorehabilitation.^4^ rTMS is a noninvasive brain stimulation technique that generates magnetic pulses to induce electric currents in the brain, modulating neural activity and neurobiological responses.^5^ Many studies^6^^–^^8^ have reported improvement in upper limb motor function following rTMS. A recent meta-analysis^9^ found a mean increase of 1.87 points in the Fugl-Meyer assessment for upper extremity (FMA-UE) compared with sham stimulation (95% CI: 0.88 to 2.86, p<0.001), supporting its clinical relevance in post-stroke rehabilitation. Although some have hypothesized that neuromodulation should be time-locked to specific behavioral tasks to maximize Hebbian plasticity,^10^ in clinical practice, rTMS is more commonly applied as a priming intervention prior to rehabilitation.^11^ However, the underlying neurophysiological mechanisms—such as changes in cortical connectivity or plasticity—remain underexplored.^12^

The interhemispheric imbalance model serves as the principal theoretical basis for applying rTMS in post-stroke rehabilitation.^13^ This model proposes that both hemispheres exert equal inhibitory influence on each other in the healthy brain. However, after a stroke, reduced inhibition and excitability in the affected hemisphere lead to increased excitability in the unaffected hemisphere, which in turn exerts a stronger inhibitory influence on peri-lesional areas. This interhemispheric imbalance may hinder functional recovery.^14^ rTMS applied to one hemisphere has been shown to influence cortical excitability in the contralateral hemisphere, possibly through modulation of interhemispheric communication; however, the underlying neural mechanisms remain unclear.^15^^,^^16^

Interhemispheric inhibition (IHI), a physiological marker of interhemispheric balance, can be assessed using paired-pulse transcranial magnetic stimulation (ppTMS), as first demonstrated by Ferbert et al.^17^ Since then, numerous studies have investigated changes in IHI after stroke^18^^,^^19^ and its modulation by rTMS,^20^^–^^22^ yet findings remain inconsistent. One major limitation is that IHI from the contralesional to the ipsilesional hemisphere in patients with severe motor impairment or corticospinal tract damage is often difficult to measure. As a result, some studies assess only unidirectional IHI^20^^–^^22^ or include only mildly affected patients with stroke,^21^ introducing selection bias. Moreover, few studies^23^^,^^24^ have integrated complementary physiological markers to validate IHI modulation, further limiting the interpretability and generalizability of current evidence.

Functional near-infrared spectroscopy (fNIRS) is increasingly used in clinical research in patient populations^25^^,^^26^ due to its noninvasive nature, portability, and tolerance to movement. It enables assessment of cortical hemodynamics during both task performance and resting state, making it suitable for studying brain function and neural networks in naturalistic settings. fNIRS offers a valuable complement to paired-pulse TMS by addressing several limitations, such as procedural complexity, time consumption, and the restriction of IHI measurements to interhemispheric interactions between bilateral primary motor cortex (M1). It enables broader investigation of interhemispheric interactions across multiple cortical regions and can be applied across patients with varying degrees of motor impairment.^27^^,^^28^ The integration of fNIRS and paired-pulse TMS enables a comprehensive assessment of cerebral hemodynamics and cortical excitability, offering insights into neurophysiological changes during stroke recovery.

This study explores the neural mechanisms of rTMS-induced post-stroke motor recovery using a multimodal approach integrating fNIRS and paired-pulse TMS, with a focus on interhemispheric interactions within motor-related cortical regions. Our hypotheses were as follows: (1) low-frequency rTMS over the contralesional M1 would result in greater upper limb motor recovery than sham stimulation in patients with stroke; (2) interhemispheric inhibition and corticospinal excitability between the bilateral M1 cortices would become more balanced after rTMS, characterized by reduced inhibition from the contralesional to the ipsilesional M1, increased inhibition in the opposite direction, enhanced excitability of the ipsilesional M1, and reduced excitability of the contralesional M1; (3) functional connectivity between bilateral motor-related cortical regions would be reduced after rTMS and; (4) changes in motor-related neurophysiological measures would be associated with improvements in upper limb motor function.

## 2 Methods

### 2.1 Participants

This is a prospective study with a randomized, sham-controlled, and single-blinded design. Twenty-nine patients with hemiparetic stroke were consecutively recruited from the Department of Rehabilitation Medicine, Shanghai Ruijin Hospital, with a median age of 70.0 years (IQR = 10.5) and 79.3% male. The diagnosis was made by clinical features and confirmed by CT and MRI. Inclusion criteria were (1) first-ever unilateral ischemic or hemorrhagic stroke; (2) between 2 weeks and 1 year after stroke onset; (3) Brunnstrom staging of hemiplegic upper limb and hand between 4 and 5; and (4) adequate cognitive function, defined as a mini-mental state examination score greater than 20 (maximum score = 30). Participants presenting with the following conditions were excluded from the study: (1) history of seizures; (2) having other neurological or severe chronic diseases; (3) severe aphasia or hemispatial neglect that could affect the participant’s ability to comply with study procedure; (4) use of drugs that might interfere on cortical excitability; and (5) having any contraindications to TMS and/or fNIRS.

**Fig. 1:**
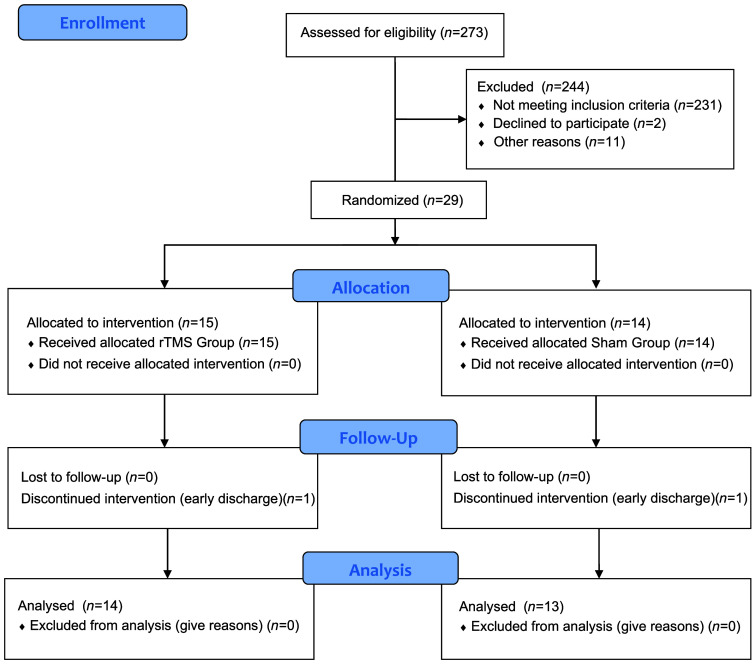
Flowchart of the trial. rTMS: repetitive transcranial magnetic stimulation. The CONSORT flow diagram illustrates recruitment, group allocation, follow-up, and analysis.

Enrolled patients were randomly assigned in a 1:1 ratio (block size = 2) to either the low-frequency (LF, 1 Hz) rTMS group or the control (sham) rTMS group. Randomization was stratified based on stroke duration (<3 months versus ≥3 months), reflecting the transition from the subacute to the later stages of stroke recovery. Group allocation was carried out by a research assistant who was not involved in the intervention or assessment procedures. The study protocol received approval from the Institutional Ethics Review Board at Shanghai Ruijin Rehabilitation Hospital, Shanghai, China. The clinical trial was pre-registered in the Chinese Clinical Trial Registry (ChiCTR) under the registration number ChiCTR2300070578. Before enrolling in the study, all participating patients provided informed consent. The participants’ flowchart is shown in Fig. 1.

### 2.2 Intervention

All subjects underwent 10-day courses of either real or sham rTMS intervention. The rTMS intervention utilized a MagTD 60 TMS device (Yi Ruide Company, Wuhan, China) equipped with a 90-mm figure-of-eight coil. The interventions were conducted in a quiet room under the supervision of a trained research staff member. Patients in the LF-rTMS group received stimulation at 1 Hz with an intensity of 100% resting motor threshold (RMT), delivering 1200 pulses per session. A total of 10 sessions were administered over 2 weeks (5 consecutive days per week). The sham group received rTMS with identical parameters (noise, duration, and frequency) to the LF-rTMS group, delivered over the M1 of the contralesional hemisphere. The coil was positioned at the same site but tilted 90 deg in the axial plane with one edge in contact with the scalp, minimizing cortical activation while maintaining similar clicking sounds.^29^

rTMS or sham rTMS interventions were administered prior to standard rehabilitation, which included 120 min of daily physical and occupational therapy over 10 sessions. Rehabilitation focused on improving limb function, posture, coordination, and basic activities of daily living. All participants also received standard pharmacological treatments, including antiplatelet agents, statins, antihypertensive drugs, neuroprotective agents, and circulation-improving medications.

### 2.3 Measurements

Outcome assessments were conducted by an independent rehabilitation physician who was not involved in the rTMS interventions. Assessments were performed at three time points: pre-treatment (T0, within 24 h before the first session), mid-treatment (T1, within 24 h after the fifth session), and post-treatment (T2, within 24 h after the final session).

#### 2.3.1 Primary outcome measure

##### Contralesional-to-ipsilesional Interhemispheric inhibition (${\mathrm{IHI}}_{\mathrm{ipsi}}$)

Contralesional-to-ipsilesional interhemispheric inhibition (${\mathrm{IHI}}_{\mathrm{ipsi}}$), representing the transcallosal suppression of the contralesional hemisphere over the ipsilesional hemisphere, was measured using a paired-pulse TMS paradigm. The conditioning stimulus (CS) was applied over the contralesional M1 and the test stimulus (TS) over the ipsilesional M1. The interstimulus interval between CS and TS was 10 ms. Both CS and TS intensities were set to elicit a 0.50 mV motor-evoked potential (MEP) amplitude. If this amplitude could not be obtained, the intensity was adjusted to 120% of the RMT.^30^

The center point of each coil was positioned over the individual M1 hotspot, with the TS coil handle oriented 45 deg to the sagittal plane and the CS coil at 90 deg. Ten TS-only and ten CS + TS trials were delivered in random order, with at least 5 s between stimuli. ${\mathrm{IHI}}_{\mathrm{ipsi}}$ was calculated as the ratio of the mean MEP amplitude in the CS + TS condition to that in the TS-only condition,^14^ such that a larger ratio indicates a smaller inhibitory effect. In cases where no MEPs could be elicited from the ipsilesional hemisphere, measurement of ${\mathrm{IHI}}_{\mathrm{ipsi}}$ was not feasible.^31^

#### 2.3.2 Secondary outcome measures

The secondary outcomes included indicators of interhemispheric imbalance, cortical excitability, and connectivity, as well as clinical assessments of upper limb motor functioning and activities of daily living (ADL). Neurophysiological indicators comprised TMS-based measures of ipsilesional-to-contralesional interhemispheric inhibition (${\mathrm{IHI}}_{\text{contra}}$), RMT, and laterality quotient (LQ), together with fNIRS-derived functional connectivity (FC). Clinical assessments included the FMA-UE, action research arm test (ARAT), modified Barthel index (MBI), and grip strength.

##### TMS measurements

###### Ipsilesional-to-contralesional interhemispheric inhibition (${\mathrm{IHI}}_{\text{contra}}$)

Ipsilesional-to-contralesional interhemispheric inhibition (${\mathrm{IHI}}_{\text{contra}}$) was assessed using the same paired-pulse TMS paradigm as ${\mathrm{IHI}}_{\mathrm{ipsi}}$, but with reversed stimulation order (CS over the ipsilesional M1 and TS over the contralesional M1). The CS and TS intensities were set to produce a 0.50 mV MEP amplitude or, if unattainable, to 120% RMT. In cases where no measurable MEP could be evoked from the ipsilesional hemisphere, the CS intensity was matched to the contralesional threshold (0.50 mV or 120% RMT).^31^

###### Resting motor threshold

Motor cortical excitability was evaluated by single pulse TMS in the contralesional and ipsilesional hemispheres of all patients at three evaluation time points. The optimal stimulation site (“hot spot”) was identified as the location over the motor cortex that consistently elicited the largest MEP in the contralateral first dorsal interosseous (FDI) muscle at rest. Subsequent neurophysiological assessments used the same “hot spot” (usually primary motor cortex, M1). The RMT was defined as the minimal stimulus intensity eliciting a MEP response of at least 50  μV amplitude with the resting FDI muscle during 5 out of 10 consecutive stimulations.^32^ If the MEP was not evocable, RMT was set to 100.

###### Laterality quotient

The LQ was calculated as [(contralesional side RMT − ipsilesional side RMT)/(contralesional side RMT + ipsilesional side RMT)] × 100 to assess the interhemispheric balance of corticospinal excitability. In this metric, values close to zero indicate symmetry, whereas deviations from zero reflect imbalance. A positive LQ reflects lower RMT (i.e., higher excitability) in the ipsilesional hemisphere, whereas a negative LQ reflects higher excitability in the contralesional hemisphere. To further capture the magnitude of imbalance irrespective of direction, the absolute value of LQ (|LQ|) was also computed.

##### fNIRS measurements

**Fig. 2:**
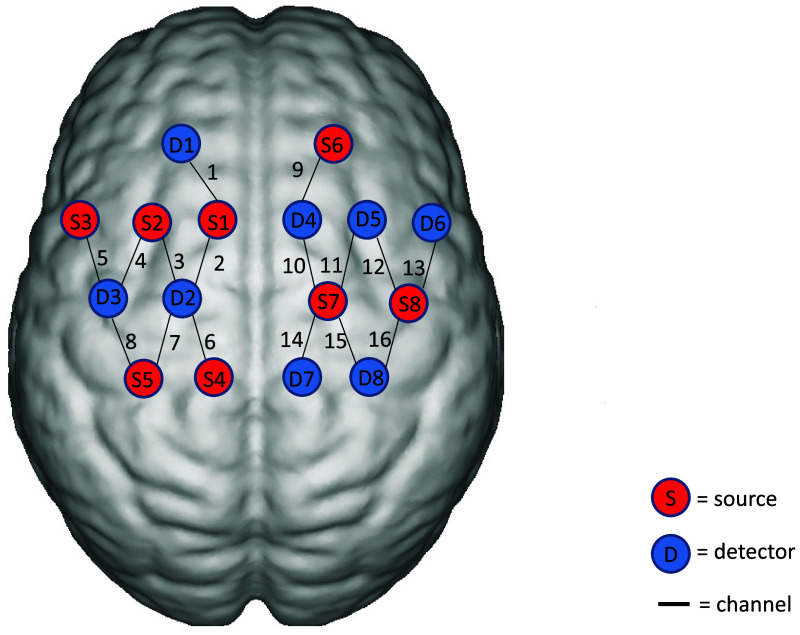
Configuration of light sources, detectors, and measurement channels. The red dots represent the light sources, and the blue dots represent the detectors. Each pair of a light source and a detector forms a channel. A total of eight light sources and eight detectors were used, forming 16 channels, measuring bilateral M1, SMA, and the PMC. Landmarks: S8: C4; D3: C3 (according to the international 10/20 system) (MNI coordinates of the optodes are available in Table S2 in the Supplementary Material.).

All fNIRS recordings were performed at least 1 h apart from rTMS to avoid acute stimulation effects. The data acquisition used a multichannel continuous wave near-infrared brain functional imaging device (NirScan-6000Br, HuiChuang, China) with wavelengths of 730, 808, and 850 nm. The sampling rate is 11 Hz. Eight light sources and eight detectors were used to form 16 effective channels; the average distance between the source and the detector is 3 cm (range 2.7 to 3.3 cm), with reference to the international 10/20 system for positioning. To investigate motor-related functional connectivity, bilateral Brodmann areas BA4 (primary motor cortex, M1) and BA6 (supplementary motor area and premotor cortex, SMA and PMC) were defined as regions of interest (ROIs) (Fig. 2). The BA4 region is involved in motor execution, whereas the BA6 region contributes to motor planning and motor learning.^33^ Resting-state fNIRS data were recorded while participants sat quietly in a separate, dimly lit room. Each participant was instructed to remain seated with eyes closed, avoid head movement, stay awake, and minimize external noise or distraction. The recording lasted for 10 min.

Preprocessing of the fNIRS raw data was conducted in MATLAB 2017b using the HOMER2 software package. First, optical intensity time series were visually inspected to evaluate the coupling quality between the photodiodes and the scalp, and channels exhibiting poor signal quality were identified and excluded from subsequent analyses. In addition, the exclude time function was used to manually remove data segments with excessive noise. To avoid unstable signals at the beginning of the recording and potential signal drifts toward the end, only data within the time window from 10 to 610 s after recording onset were retained for further analysis.^34^ Second, raw optical intensity data from 16 channels were converted into optical density (OD) using the modified Beer–Lambert law.^35^ Third, because the coefficient of variation is highly sensitive to extreme values introduced by motion artifacts, signal quality was reassessed after artifact correction, with a threshold set at 0.25.^36^ Fourth, motion artifacts were detected and corrected using the hmrMotionArtifactByChannel and hmrMotionCorrectSpline functions, with the following parameters: tMotion = 0.5, tMask = 2, STDEVthresh = 20, AMPthresh = 0.5, and pSpline = 0.99.^37^^,^^38^ Fifth, the optical density data were further subjected to band-pass filtering to reduce physiological noise of nonneuronal origin. Specifically, the hmrBandpassFilt function implemented in the HOMER2 software package was applied, with a passband of 0.01 to 0.10 Hz. This frequency range was selected to preserve low-frequency components associated with resting-state hemodynamic fluctuations while effectively attenuating high-frequency physiological noise such as cardiac pulsations (≈1  Hz), respiratory rhythms (≈0.2 to 0.3 Hz), and blood pressure–related oscillations, as well as minimizing very low-frequency signal drifts.^39^ Sixth, the band-pass-filtered optical density data were converted into changes in hemoglobin concentration, including oxygenated hemoglobin (HbO) and deoxygenated hemoglobin (HbR), using the modified Beer–Lambert law.^40^

Given that resting-state fNIRS signals may contain substantial systemic physiological fluctuations (e.g., scalp blood flow and global hemodynamic oscillations), motion artifact correction and band-pass filtering alone may be insufficient to fully remove extracerebral contributions. To further enhance the robustness of functional connectivity analyses, a principal component analysis (PCA)-based global physiological noise modeling and regression procedure was applied following the aforementioned preprocessing steps. Specifically, after obtaining hemoglobin concentration signals, the multichannel time series of HbO and HbR were first mean-centered across time, and PCA was then performed separately for each hemoglobin type. The first principal component (PC1), which explained the largest proportion of variance, was extracted and treated as a proxy for global systemic physiological fluctuations, primarily reflecting extracerebral blood flow and nonneuronal global oscillations. Subsequently, this PC1 component was regressed out from the HbO and HbR time series of each channel using linear regression while preserving each channel’s mean level. By explicitly modeling and removing global physiological noise, this approach reduces the potential influence of systemic covariation on functional connectivity estimates.^39^

Finally, after PCA-based regression, signals were further aggregated at the ROI level, with each ROI time series computed as the average of its corresponding channel signals. Pearson correlation coefficients were then calculated between ROI-level HbO and HbR time series to quantify resting-state functional connectivity between ROIs. To enable inter-subject comparison and simplify lateralized analysis, data from left-hemisphere stroke patients were mirrored across the sagittal plane, such that the lesioned hemisphere was consistently represented on the right side throughout all analyses. The selected ROIs were the contralesional BA6 (channels 1 to 5), the contralesional BA4 (channels 6 to 8), the ipsilesional BA6 (channels 9 to 13), and the ipsilesional BA4 (channels 14 to 16). (see Table S1 in the Supplementary Material for full channel anatomical details).

##### Clinical assessments

###### Fugl-Meyer assessment of the upper extremity

The FMA-UE assesses 33 items on a three-point scale (0 for complete paralysis to 66 for normal motor function). It includes shoulder and elbow components, covering upper limb reflex activity, flexor and extensor synergy movements, isolated joint movements, and normal reflex activity. It also evaluates wrist stability, hand movement, coordination, and speed.

###### Action research arm test

The ARAT comprises 19 test items, each assessed on a four-level scale, resulting in a total score range of 0 to 57 for the affected upper limb. Higher scores indicate better activity. ARAT evaluates an individual’s ability to manipulate objects of different sizes, weights, and shapes, serving as a standardized rating scale for post-stroke upper limb and hand function.

###### Modified Barthel index

The MBI was documented, encompassing personal hygiene, self-bathing, feeding, using the toilet, stair climbing, getting dressed, bowel control, bladder control, chair/bed transfer, and ambulation. The total score on the MBI scale is 100 points.

###### Grip strength

Grip strength was measured with a hand dynamometer. Participants were directed to squeeze the handgrip maximally for 5 s, undergoing three attempts with at least a 60-s rest between each trial. The highest recorded grip strength in kilograms was utilized for analysis.

### 2.4 Statistical Analysis

Statistical analysis was performed using IBM SPSS Statistics 26.0 and R version 4.5.0 (R Foundation for Statistical Computing), with statistical significance defined as p<0.05 (two-tailed).

To compare baseline characteristics between the two groups, categorical variables (e.g., gender, hypertension, and stroke type) were analyzed using Fisher’s exact test due to the small sample size and low expected cell counts. Continuous variables (e.g., age) were assessed for normality using the Shapiro–Wilk test. If the data were normally distributed, group comparisons were performed using independent samples t-tests; otherwise, the Mann–Whitney U test was applied.

For outcome measures, a Shapiro–Wilk test was first conducted to assess the normality of all outcome variables. The normality assumption was satisfied for most variables, including IHI, iRMT, LQ, |LQ|, FMA-UE, and ADL, whereas FC and ARAT scores violated this assumption. Grip strength data and cRMT were log-transformed using the formula $\log_{10}$ (x+1) to improve normality prior to analysis. For variables conforming to normality, a series of two-way mixed-design ANOVAs was performed to examine the effects of group (rTMS versus sham) and time (pre-, mid-, post-intervention), with time treated as a within-subject factor and group as a between-subject factor. Mauchly’s test was used to assess the sphericity assumption; if violated, Greenhouse–Geisser corrections were applied to the degrees of freedom. Upon finding a significant interaction effect, repeated-measures ANOVAs were subsequently performed within each group to assess time effects. When significant, Bonferroni-corrected post hoc pairwise comparisons were conducted to identify specific time point differences. For the FC and ARAT scores, which did not meet the normality assumption, a nonparametric aligned rank transform (ART) ANOVA was used to evaluate the same effects. Upon finding a significant interaction effect, Friedman’s test was subsequently performed to assess time effects within each group. When significant, Bonferroni-corrected post hoc pairwise Wilcoxon signed-rank comparisons were conducted to identify specific differences between time points. In addition, to determine whether the changes from baseline (ΔT1−T0 and ΔT2−T0) differed between groups, independent-samples t-tests were used for normally distributed data, and Mann-Whitney U tests were applied when normality was not assumed.

The ${\mathrm{IHI}}_{\mathrm{ipsi}}$ was defined as the primary outcome, and no multiplicity correction was applied to this pre-specified endpoint. For secondary outcomes, false discovery rate (FDR) correction was applied to the group × time interaction effects within each outcome domain (neurophysiological, connectivity, and clinical) to control for multiple comparisons.

Correlation analyses were performed to examine the associations between changes in neurophysiological indices (ΔIHI, ΔLQ, ΔRMT, ΔFC) and ΔFMA-UE, as well as among neurophysiological measures themselves. Pearson’s or Spearman’s coefficients were applied according to normality. Multiple comparisons were corrected using the FDR method. As LQ was derived from RMT, their correlation was not analyzed to avoid circular dependence.

## 3 Results

Twenty-seven patients completed the interventions without encountering incidents, severe side effects, or discomfort, demonstrating the well-tolerated nature of the procedure (Fig. 1). There were no statistical differences between the two groups in gender, age, onset time, stroke type, injury site, and functional score (p>0.05, Table 1). Lesion characteristics of the stroke participants are provided in Table S3 in the Supplementary Material.

**Table 1:** Baseline characteristics (n=27).

Characteristic	Real rTMS (n=14)	Sham rTMS (n=13)	p
Age, median (IQR), years	68.5 (9.5)	72.0 (18.0)	0.616^a^
Gender, n (%)			0.385^b^
Male	12 (85.7)	9 (69.2)	
Female	2 (14.3)	4 (30.8)	
Lesion location, n (%)			—
Cortical	2 (14.3)	0 (0)	
Subcortical	8 (57.1)	9 (69.2)	
Cortical-subcortical	4 (28.6)	4 (30.8)	
Stroke type, n (%)			0.596^b^
Ischemic	13 (92.9)	11 (84.6)	
Hemorrhagic	1 (7.1)	2 (15.4)	
Side of lesion, n (%)			0.252^b^
Left	8 (57.1)	4 (30.8)	
Right	6 (42.9)	9 (69.2)	
Time from stroke, median (IQR), months	2.6 (1.9)	2.6 (4.1)	0.905^a^
Hypertension, n (%)	12 (85.7)	13 (100)	0.481^b^
Diabetes, n (%)	4 (28.6)	7 (53.8)	0.252^b^
Right-hand dominance, n (%)	14 (100)	13 (100)	—
BRS-UE, n (%)			0.209^b^
Stage 4	12 (85.7)	8 (61.5)	
Stage 5	2 (14.3)	5 (38.5)	
BRS-hand, n (%)			1.000^b^
Stage 4	9 (64.3)	8 (61.5)	
Stage 5	5 (35.7)	5 (38.5)	
FMA-UE, mean (*SD*)	36.4 (12.7)	39.7 (15.2)	0.549^c^
ARAT, median (IQR)	22.0 (30.8)	36.0 (37.5)	0.715^a^
MEPs (+), n (%)	10 (71.4)	10 (76.9)	1.000^b^

*Abbreviations*: rTMS, repetitive transcranial magnetic stimulation; IQR, interquartile range; SD, standard deviation; BRS-UE, Brunnstrom staging of the upper extremity; BRS-hand, Brunnstrom staging of the hand; FMA-UE, Fugl–Meyer assessment of the upper extremity; ARAT, action research arm test; MEPs (+), presence of motor evoked potentials in the ipsilesional hemisphere.

No statistical comparisons were conducted for lesion location and hand dominance due to limited variability and small cell sizes.

a p values are calculated by the Mann–Whitney U test.

b p values are calculated by Fisher’s exact test.

c p values are calculated by independent samples t-tests.

### 3.1 Result of Primary Outcome

**Fig. 3:**
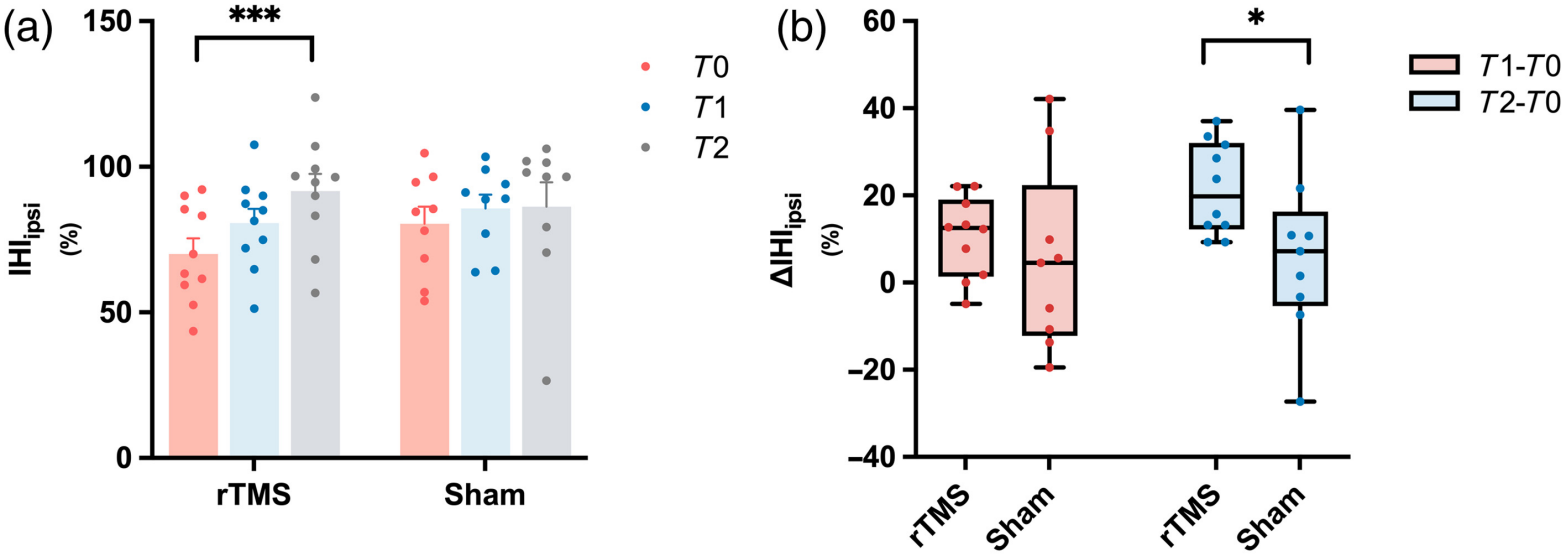
Changes in ${\mathrm{IHI}}_{\mathrm{ipsi}}$ in rTMS and sham groups. (a) ${\mathrm{IHI}}_{\mathrm{ipsi}}$ at three time points (T0, T1, T2) in the rTMS and sham groups; note that higher IHI values indicate weaker interhemispheric inhibition. (b) Changes in ${\mathrm{IHI}}_{\mathrm{ipsi}}$ from baseline (ΔT1−T0, ΔT2−T0) across groups. Error bars represent SEM.*p<0.05, ***p<0.001.

In this study, a total of eight baseline MEP (-) patients, in whom contralesional-to-ipsilesional interhemispheric inhibition (${\mathrm{IHI}}_{\mathrm{ipsi}}$) could not be detected, were excluded from this analysis. A mixed-design ANOVA revealed a significant main effect of time on ${\mathrm{IHI}}_{\mathrm{ipsi}}$ [F(2,34)=7.304, p=0.002], with no significant group or interaction effects. In the TMS group, repeated-measures ANOVA showed a significant time effect [F(2,18)=19.880, p<0.001], with post hoc comparisons revealing significant increases in ${\mathrm{IHI}}_{\mathrm{ipsi}}$ from T0 to T2 (p<0.001) and from T1 to T2 (p=0.017). No significant changes were observed in the sham group (p=0.625) [Fig. 3(a)]. Between-group comparisons showed a significantly greater increase in ${\mathrm{IHI}}_{\mathrm{ipsi}}$ from baseline to post-intervention (ΔT2−T0) in the rTMS group compared to sham (p=0.048) [Fig. 3(b)].

### 3.2 Result of Secondary Outcome

#### 3.2.1 TMS results

For ipsilesional-to-contralesional IHI (${\mathrm{IHI}}_{\text{contra}}$), mixed-design ANOVA revealed no significant main or interaction effects (all p>0.05), indicating comparable ${\mathrm{IHI}}_{\text{contra}}$ values between the rTMS and sham groups across sessions.

**Fig. 4:**
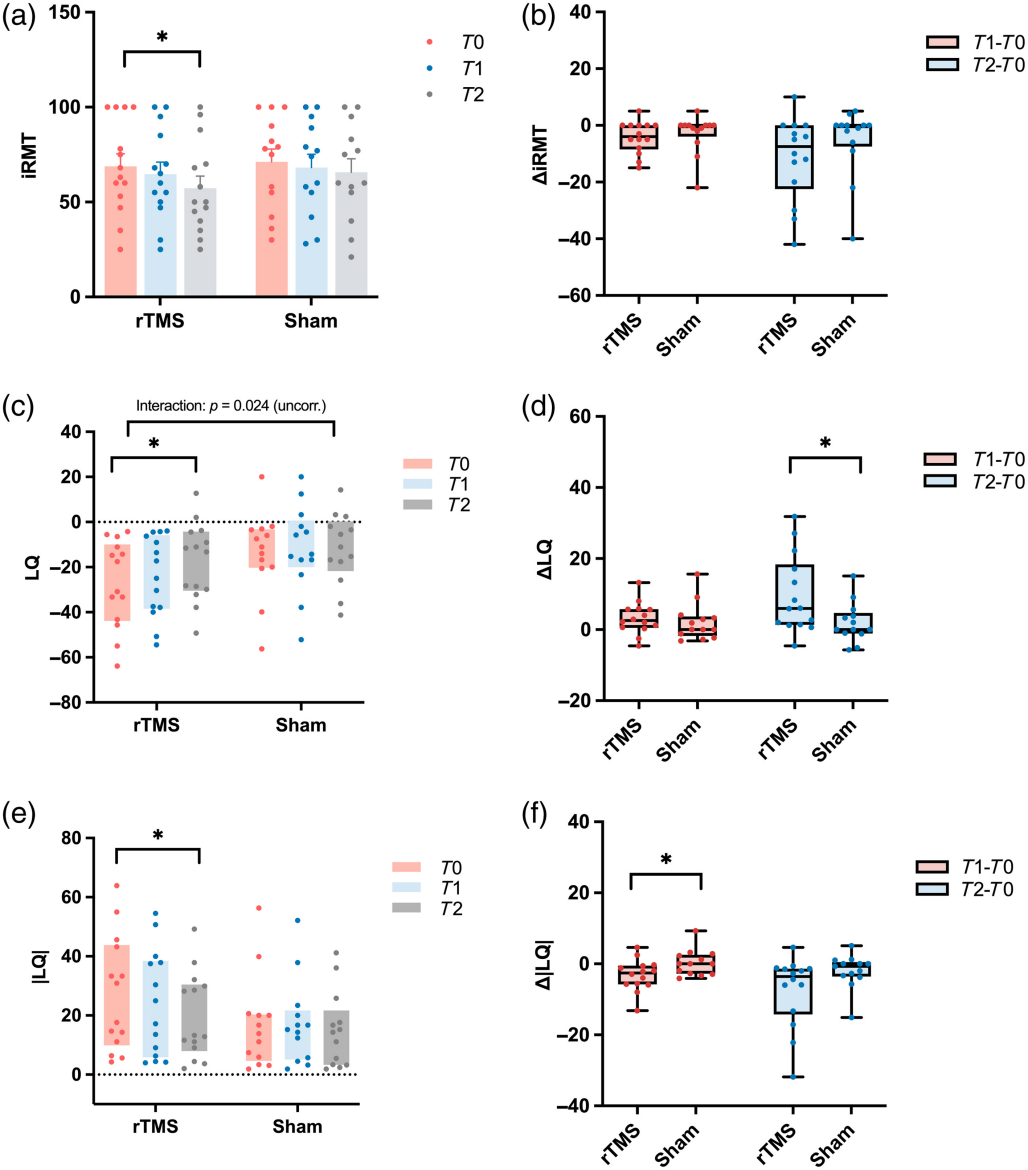
Changes in iRMT, LQ, and |LQ| in rTMS and sham groups. (a) iRMT at three time points (T0,T1,T2) in the rTMS and sham groups; (b) changes in iRMT from baseline (ΔT1−T0, ΔT2−T0) across groups; (c) LQ at three time points in both groups, showing a group × time interaction (p=0.024, uncorrected; FDR-corrected p=0.096); (d) changes in LQ from baseline (ΔT1−T0, ΔT2−T0) across groups; (e) |LQ| at three time points (T0,T1,T2) in the rTMS and sham groups; (f) changes in |LQ| from baseline (ΔT1−T0, ΔT2−T0) across groups. Error bars represent SEM. *p<0.05.

The analysis also showed a significant main effect of time for ipsilesional resting motor threshold (iRMT) [F(1.203,60.165)=9.226, p=0.003], with no significant group or interaction effects. In the rTMS group, iRMT significantly decreased over time [F(2,26)=7.850, p=0.002], with reductions from T0 to T2 (p=0.035) and from T1 to T2 (p=0.047), whereas no significant change was observed in the sham group [Fig. 4(a)]. Between-group comparisons of iRMT change (ΔT1−T0 and ΔT2−T0) were not significant [Fig. 4(b)]. By contrast, contralesional resting motor threshold (cRMT) did not exhibit any significant main or interaction effects.

Similarly, LQ analysis revealed a significant main effect of time [F(1.381,34.536)=9.451, p=0.002], whereas the time × group interaction [F(1.381,34.536)=4.829, p=0.024] did not remain significant after FDR correction ($p_{\mathrm{adj}}=0.096$). Post hoc analysis showed that in the rTMS group, LQ significantly increased from T0 to T2 (p=0.018) and from T1 to T2 (p=0.024), whereas no significant change was found in the sham group [Fig. 4(c)]. This increase in LQ reflects a shift of corticospinal excitability toward the ipsilesional hemisphere, indicating reduced contralesional dominance or even ipsilesional predominance. Between-group comparison further revealed that LQ improvements from baseline to post-intervention (ΔT2−T0) were significantly greater in the rTMS group compared to the sham [p=0.034; Fig. 4(d)]. Consistently, the analysis of |LQ| showed a significant between-group difference during the early phase (ΔT1−T0, p=0.036), indicating a greater reduction of interhemispheric asymmetry in the rTMS group compared with the sham group, whereas the difference from baseline to post-intervention (ΔT2−T0, p=0.058) showed a trend toward significance [Figs. 4(e) and 4(f)].

#### 3.2.2 fNIRS results

**Fig. 5:**
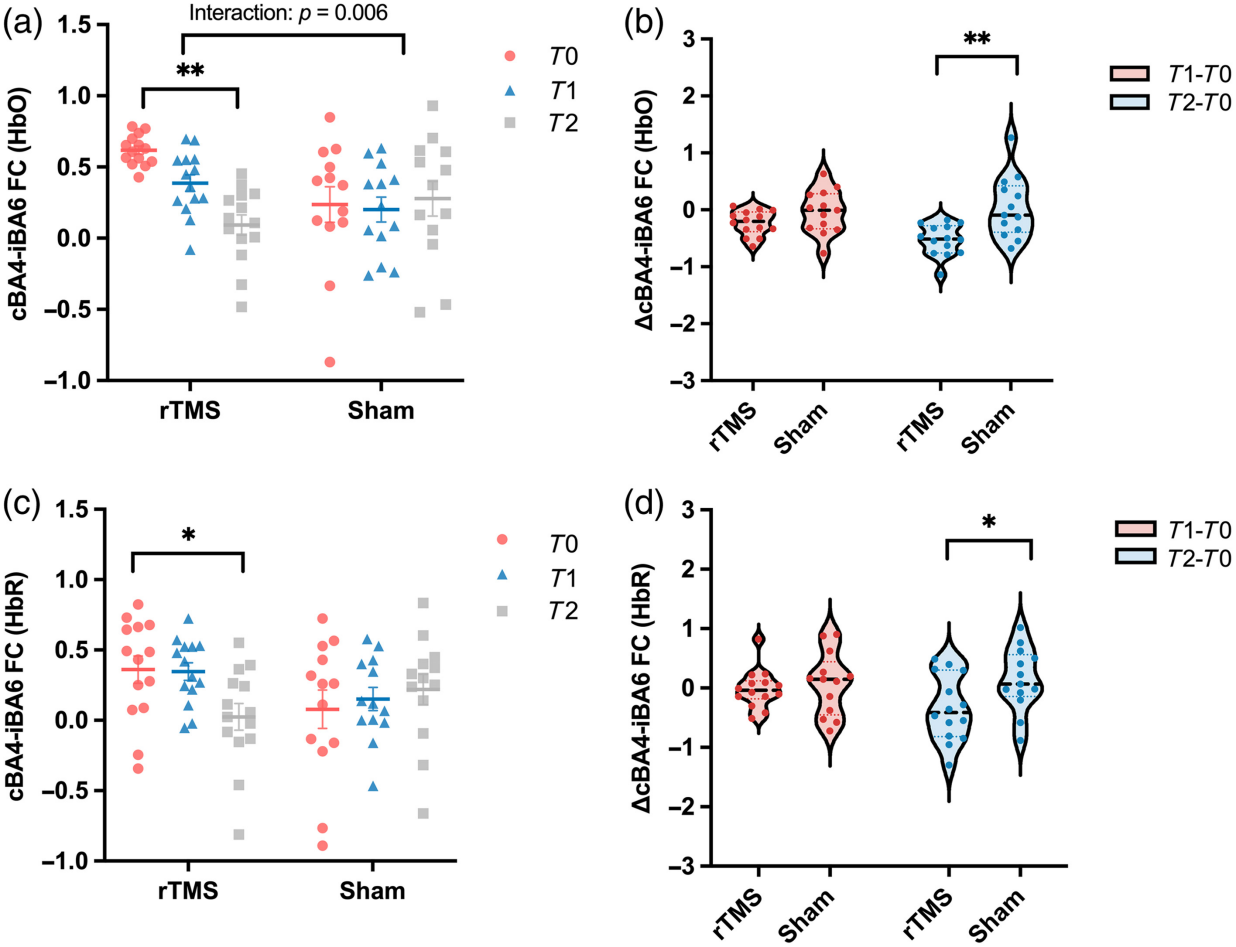
Changes in cBA4-iBA6 FC in rTMS and sham groups. (a) cBA4-iBA6 FC (HbO) at three time points (T0,T1,T2) in both groups, showing a significant group × time interaction (p=0.001, uncorrected; FDR-corrected p=0.006); (b) changes in cBA4-iBA6 FC (HbO) from baseline (ΔT1−T0, ΔT2−T0) across groups; (c) cBA4-iBA6 FC (HbR) at three time points (T0,T1,T2) in both groups; (d) changes in cBA4-iBA6 FC (HbR) from baseline (ΔT1−T0, ΔT2−T0) across groups. Error bars represent SEM. *p<0.05, **p<0.001.

**Fig. 6:**
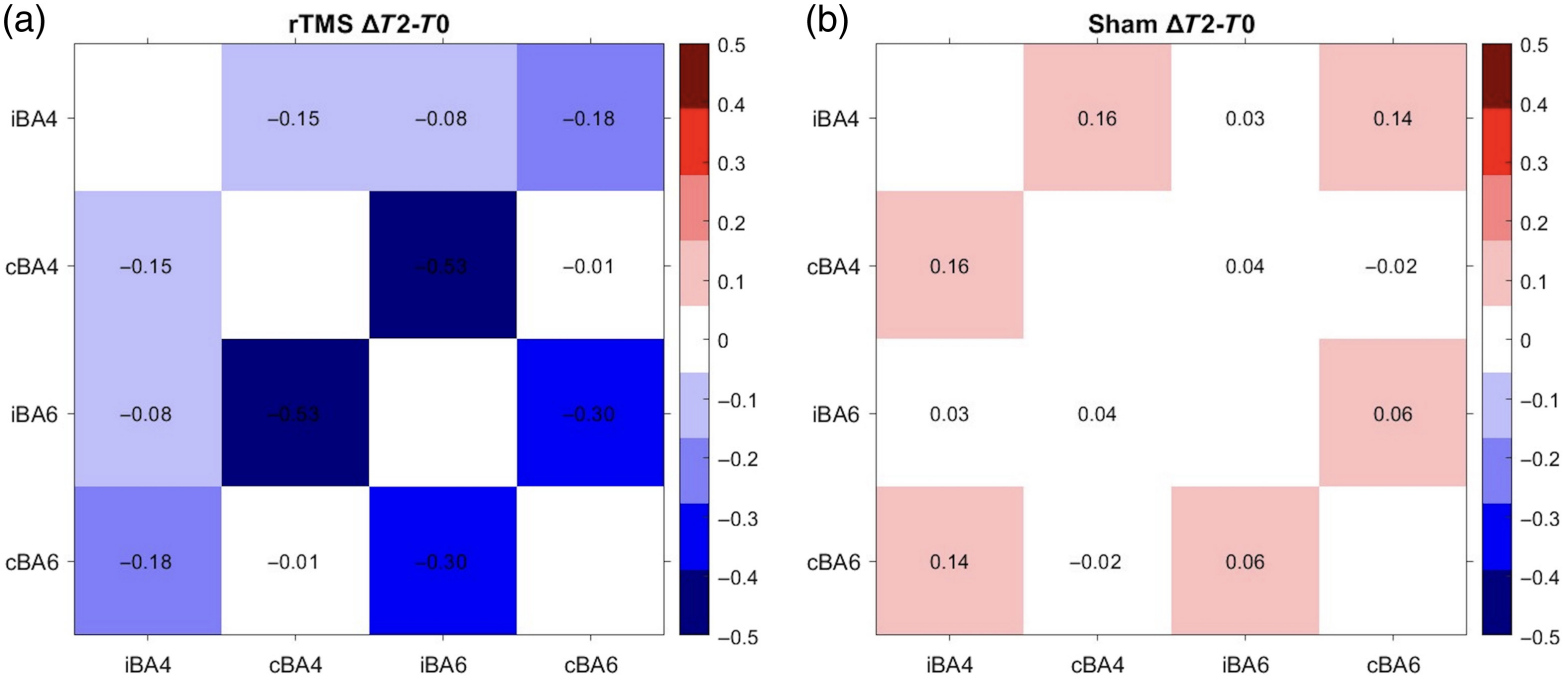
Changes in group-level functional connectivity (HbO, Pearson’s r) between bilateral motor ROIs from T0 to T2. Panels (a) and (b) show ΔT2 to T0 correlation matrices for the rTMS and sham groups, respectively. ROIs include BA4 (M1) and BA6 (PMC/SMA) in the ipsilesional (i) and contralesional (c) hemispheres. Color indicates the magnitude and direction of connectivity changes.

An ART ANOVA revealed a significant group × time interaction for the FC between contralesional BA4 and ipsilesional BA6 (cBA4-iBA6) based on HbO signals [F(2,50)=7.704, p=0.001, $p_{\mathrm{adj}}=0.006$, FDR-corrected across six ROI pairs], whereas other channels showed no significant interaction effects [Figs. 5(a) and 6]. Post-hoc analyses within the TMS group showed a significant main effect of time ($\chi^{2}(2)=20.571$, p<0.001), with a significant reduction from T0 to T2 (p=0.003). By contrast, no significant time effect or pairwise differences were found in the sham group [Fig. 5(a)]. Between-group comparison revealed a significantly greater reduction in cBA4-iBA6 connectivity from T0 to T2 in the rTMS group compared with the sham group (p=0.004) [Fig. 5(b)].

Analyses based on HbR signals showed a comparable pattern. Although none of the group × time interactions across ROI pairs reached significance after FDR correction, the cBA4-iBA6 pair followed a similar direction as the HbO effect [F(2,50)=3.895, p=0.027, $p_{\mathrm{adj}}=0.084$, FDR-corrected across six ROI pairs; Fig. 5(c)]. Within-group analysis in the rTMS group revealed a significant reduction from T0 to T2 (p=0.144), whereas no significant time effect was observed in the sham group [Fig. 5(c)]. The between-group comparison (ΔT2−T0) showed a greater reduction in cBA4-iBA6 connectivity in the rTMS group compared with the sham group [p=0.037; Fig. 5(d)].

#### 3.2.3 Motor function, grip strength, and daily living performance

**Fig. 7:**
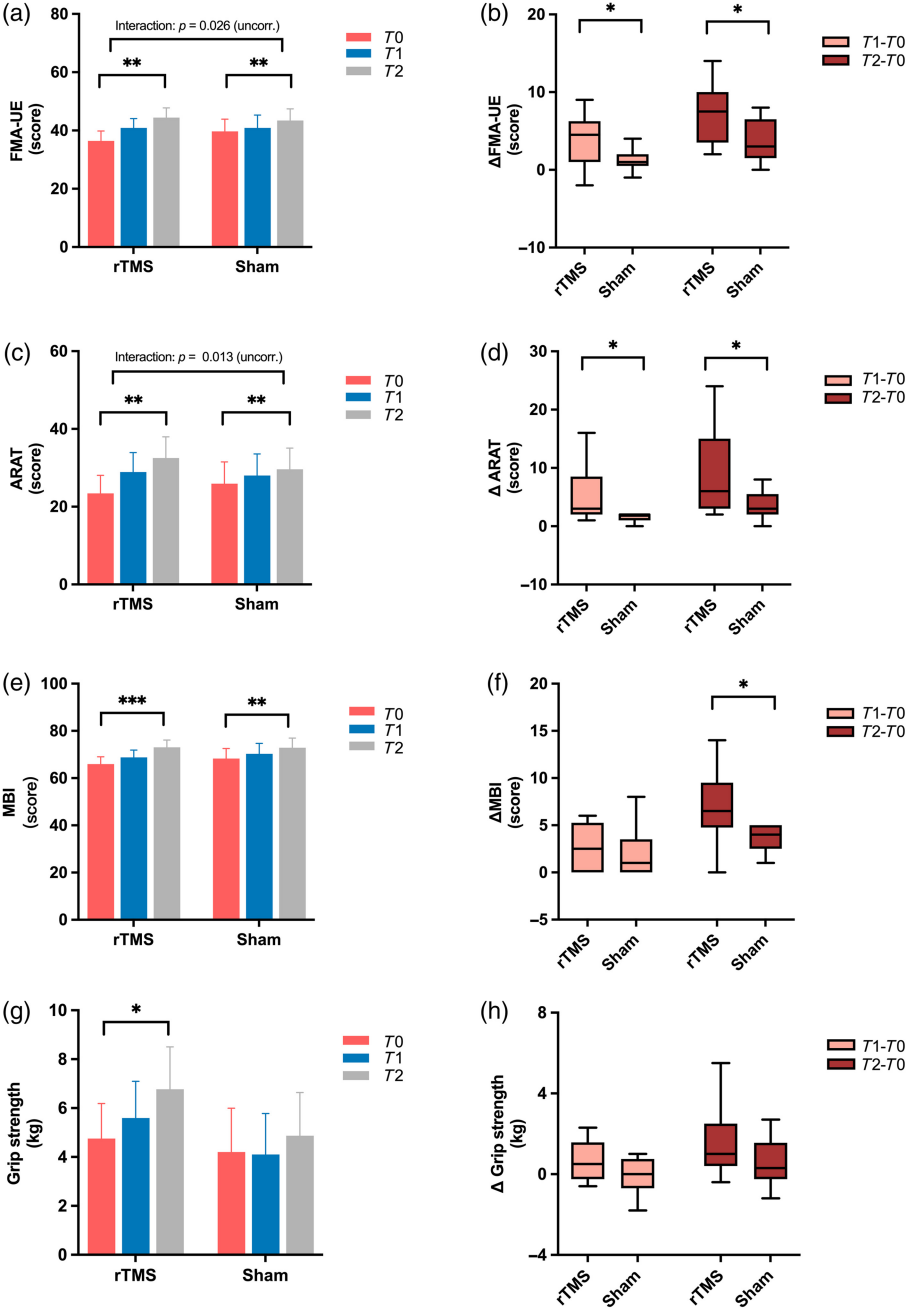
Changes in FMA-UE, ARAT, MBI, and grip strength over time in the rTMS and sham groups. (a), (b) FMA-UE scores at three timepoints and corresponding change scores (ΔT1−T0, ΔT2−T0). (c), (d) ARAT scores and change scores across time. (e), (f) MBI scores and corresponding changes from baseline. (g), (h) Grip strength (log-transformed for analysis) and change scores. Error bars represent SEM. Interaction p-values shown in panels (a) and (c) are uncorrected (p=0.026 and p=0.013, respectively); FDR-corrected p-values were 0.052 and 0.052. *p<0.05, **p<0.001, ***p<0.001.

For the FMA-UE scores, a mixed-design ANOVA revealed a significant main effect of time [F(1.385,69.256)=33.33, p<0.001] and a time × group interaction that approached significance after FDR correction [F (1.385,69.256)=4.73, p=0.026, FDR-corrected p=0.052]. Post hoc analyses showed significant improvements in both groups from T0 to T2 (rTMS: p=0.001; sham: p=0.001) and from T1 to T2 (rTMS: p<0.001; sham: p=0.020) [Fig. 7(a)]. Between-group comparisons revealed greater FMA-UE improvements in the rTMS group than in the sham group for both ΔT1−T0 (p=0.035) and ΔT2−T0 (p=0.029) [Fig. 7(b)].

For the ARAT scores, an ART ANOVA revealed a significant main effect of time [F (2, 50) = 38.457, p<0.001] and a time × group interaction that approached significance after FDR correction [F(2,50)=4.758, p=0.013, FDR-corrected p=0.052]. Follow-up Friedman tests confirmed significant changes over time in both the rTMS [$\chi^{2}(2)=28.000$, p<0.001] and sham [$\chi^{2}(2)=21.535$, p<0.001] groups. Bonferroni-corrected Wilcoxon tests showed significant improvements between all timepoints in both groups (all p<0.017) [Fig. 7(c)]. Between-group comparisons showed significantly greater ARAT improvements in the rTMS group compared with sham for both ΔT1−T0 (p=0.030) and ΔT2−T0 (p=0.028) [Fig. 7(d)].

For the MBI scores, a mixed-design ANOVA revealed a significant main effect of time [F(1520,76.017)=51.655, p<0.001], with no significant group or interaction effects. Post hoc analyses showed significant improvements at all time points in both the rTMS group (all p≤0.003) and the sham group (all p≤0.031) [Fig. 7(e)]. Between-group comparison showed a significantly greater ΔT2−T0 improvement in the rTMS group compared with sham (p=0.040) [Fig. 7(f)].

For grip strength, a mixed-design ANOVA revealed a significant main effect of time [F(2,50)=7.826, p=0.001], with no significant group or interaction effects. In the rTMS group, a significant time effect was observed [F(2,26)=7.005, p=0.004], with a significant improvement from T0 to T2 (p=0.012), whereas no significant changes were found in the sham group (p=0.114) [Fig. 7(g)]. Between-group comparisons of grip strength changes (ΔT1−T0 and ΔT2−T0) revealed no significant differences [Fig. 7(h)].

#### 3.2.4 Correlation between neurophysiology and behavior

**Fig. 8:**
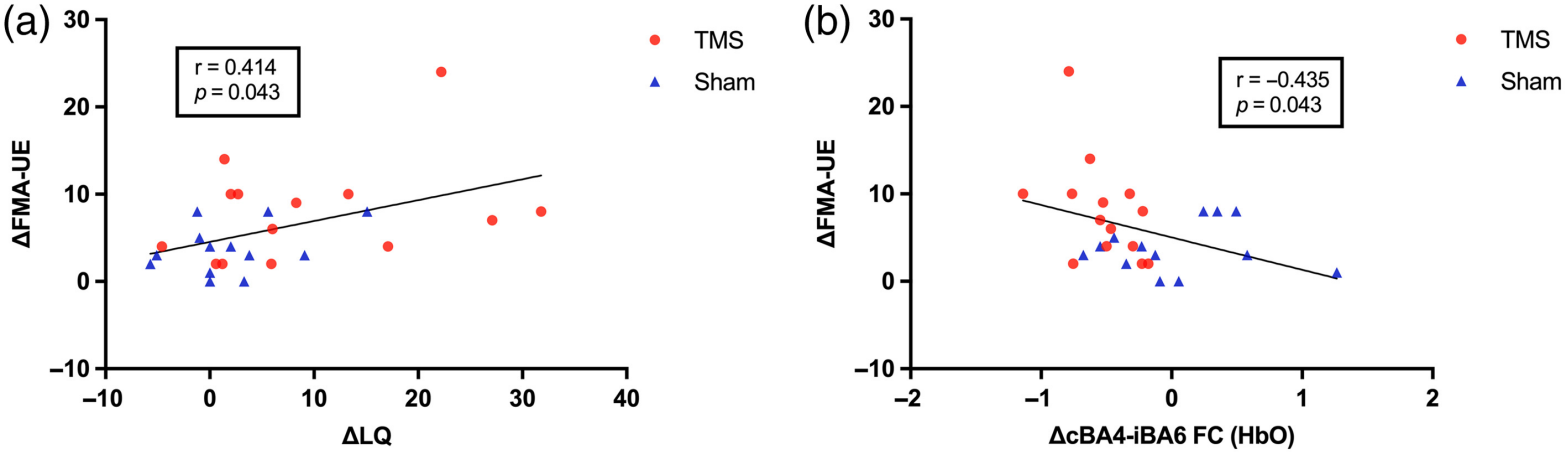
Correlations between neurophysiological and behavioral changes. (a) Significant positive correlation between ΔLQ and ΔFMA-UE (r=0.414, p=0.032, $p_{\mathrm{adj}}=0.043$). (b) Significant negative correlation between ΔcBA4-iBA6 FC (HbO) and ΔFMA-UE (r=−0.435, p=0.023, $p_{\mathrm{adj}}=0.043$).

To explore whether neurophysiological changes from baseline to post-intervention were associated with behavioral recovery, correlation analyses were performed between ΔIHIipsi, ΔLQ, ΔiRMT, and ΔcBA4-iBA6 FC (HbO) with ΔFMA-UE scores. A positive correlation was observed between ΔLQ and ΔFMA-UE [r=0.414, p=0.032, FDR-corrected p=0.043; Fig. 8(a)] and a negative correlation between ΔiRMT and ΔFMA-UE (r=−0.434, p=0.024; FDR-corrected p=0.043), indicating that a greater shift of corticospinal excitability toward the ipsilesional hemisphere and larger reductions in ipsilesional RMT were associated with greater motor improvements. In addition, a significant negative correlation was found between ΔcBA4-iBA6 FC (HbO) and ΔFMA-UE [r=−0.435, p=0.023, FDR-corrected p=0.043, Fig. 8(b)]. This relationship indicates that decreased interhemispheric cBA4-iBA6 FC was associated with greater upper limb motor improvements. Correlation analyses revealed no significant associations among the neurophysiological indices.

## 4 Discussion

In this study, we demonstrated that a 2-week course of 1 Hz rTMS applied to the contralesional M1 significantly improved upper limb motor recovery in patients with stroke. Neurophysiological changes detected by ppTMS and fNIRS suggest that rTMS may have modulated interhemispheric interactions between the stimulated contralesional M1 and the ipsilesional motor cortices, which may be associated with motor function improvement.

### 4.1 Reduction of Contralesional-to-Ipsilesional Inhibition After rTMS

Using ppTMS to assess IHI, this study found that low-frequency rTMS reduced inhibitory influence from the stimulated contralesional M1 to the ipsilesional M1. However, no significant change was observed in the reverse direction, from the ipsilesional to the contralesional M1. In line with this finding, previous studies involving noninvasive brain stimulation after stroke have reported comparable results.^20^^,^^21^^,^^41^^,^^42^ However, these rTMS studies^20^^,^^21^ typically focused only on contralesional-to-ipsilesional inhibition. By assessing IHI in both directions, our study confirms previous findings and further shows that low-frequency rTMS mainly reduces inhibition from the contralesional hemisphere, whereas inhibition in the reverse direction remains unchanged. Previous studies observed that low-frequency rTMS reduced the ipsilesional silent period (iSP) from the contralesional to the ipsilesional hemisphere compared with sham stimulation.^20^^,^^21^ Due to its relative simplicity, the iSP measurement currently prevails in the literature.^13^ However, interhemispheric inhibition induced by ppTMS or iSP methods has distinct effects on intracortical circuitry modulation^13^ and is mediated by different receptors.^43^ Therefore, these two methods should be considered as complementary rather than equivalent. Our findings, obtained through the ppTMS method, support and extend previous iSP-based observations of rTMS-induced changes in interhemispheric inhibition.

### 4.2 Reduction of Functional Interhemispheric Connectivity after rTMS

In addition to modulating IHI, rTMS may also influence broader cortical networks involved in motor recovery. Using fNIRS to assess resting-state functional connectivity (RSFC), this study found that low-frequency rTMS reduced RSFC from the stimulated contralesional M1(BA4) to the ipsilesional SMA and PMC (BA6). This finding reflects altered interhemispheric functional coupling between the bilateral motor regions. Previous studies have shown that excessive interhemispheric motor connectivity is often associated with poorer motor performance or maladaptive reorganization after stroke.^44^ Conversely, several intervention studies have reported that decreases in motor-network connectivity following therapy are accompanied by functional improvement.^45^^,^^46^ Therefore, the reduced RSFC between the contralesional M1 and the ipsilesional SMA/PMC observed here may reflect less of maladaptive interhemispheric coupling, possibly accompanied by re-engagement of perilesional motor areas. Consistent with our findings, previous studies have reported that, after stroke, reduced activation in the ipsilesional M1 is often accompanied by increased activity in adjacent motor-related areas such as PMC and SMA.^47^^,^^48^ Those changes are thought to enhance residual motor output through alternative pathways, including the reticulospinal and spared corticospinal tracts.^49^ Supporting this view, a study using TMS to inhibit the ipsilesional PMC observed reduced hand motor performance in chronic stroke patients, highlighting the compensatory role of the SMA and PMC regions in post-stroke recovery.^50^

Interestingly, although a decrease in functional connectivity between bilateral M1 was observed in the rTMS group, this change did not reach statistical significance for either time or group factors in this study. By contrast, ppTMS revealed altered IHI between these regions, suggesting that ppTMS may be more sensitive to detecting changes in interhemispheric interaction. Nonetheless, fNIRS offers valuable insights by identifying potential cortical targets beyond M1 that may be modulated by rTMS.^51^ Furthermore, previous research suggests that interhemispheric inhibition becomes more prominent and aberrantly engaged during the phases of movement preparation and early execution,^13^ rather than at rest. Given the high movement tolerance of fNIRS, future studies could utilize this technique to further explore how rTMS modulates interhemispheric interactions during active motor tasks.

### 4.3 Restoration of Bilateral M1 Excitability Balance After rTMS

In addition to evaluating changes in interhemispheric interaction, this study also calculated the LQ to assess the balance of cortical excitability between bilateral M1 regions. The LQ increased over time in the rTMS group but remained stable in the sham group, indicating a shift of excitability toward the ipsilesional hemisphere. Although iRMT alone did not exhibit a significant interaction, the trend-level change in LQ suggests that rTMS modulated corticospinal excitability in a relative rather than absolute manner. This effect appeared to be driven mainly by the reduction in ipsilesional RMT, indicating enhanced excitability of the affected hemisphere through interhemispheric disinhibition, consistent with previous findings.^21^

Although the absolute LQ (|LQ|) initially decreased, reflecting reduced interhemispheric asymmetry, further enhancement of ipsilesional excitability likely led to smaller or even reversed asymmetry in some participants. Thus, the observed pattern does not necessarily represent direct “rebalancing” between hemispheres but rather an rTMS-induced facilitation of ipsilesional excitability that secondarily modifies the interhemispheric relationship. These results suggest that low-frequency rTMS acts primarily by restoring excitability in the ipsilesional hemisphere, rather than symmetrically normalizing bilateral motor cortical activity.^16^

### 4.4 Improvements in Upper-Limb Motor Function after rTMS

Behavioral measures exhibited varying levels of sensitivity to rTMS effects. Upper limb-specific scales such as the FMA-UE and ARAT showed time × group interactions that approached significance after correction, consistent with their direct relevance to the targeted motor network.^52^ By contrast, broader or less fine-grained measures such as the MBI and grip strength primarily showed main effects of time without significant interactions. These outcomes may require more substantial or prolonged improvements to yield clear between-group differences.^20^

### 4.5 Correlation between Neurophysiology and Behavior

Importantly, we also observed relationships between changes in neurophysiological markers and upper limb motor recovery in stroke patients. Specifically, greater increases in LQ and larger reductions in iRMT were correlated with improvements in FMA-UE scores. These findings suggest that a shift of corticospinal excitability toward the ipsilesional hemisphere may accompany motor recovery after stroke, in line with previous neurophysiological evidence.^53^^–^^55^ Furthermore, changes in interhemispheric functional connectivity between cBA4 and iBA6 were also related to behavioral improvement, with decreased connectivity associated with greater gains in motor function. This observation suggests that changes in interhemispheric network interactions may accompany functional motor recovery.^56^^,^^57^ In this context, interhemispheric connectivity patterns measured with fNIRS may provide complementary physiological information for tracking recovery-related cortical reorganization following neuromodulatory interventions.

## 5 Limitations

Several limitations should be acknowledged in this study. First, the sample size was relatively small, and the study adopted a single-blind design, which may limit the generalizability and internal validity of the findings. Second, considerable heterogeneity existed in lesion location and disease duration among participants, which may have influenced individual variability in response to the rTMS intervention. Although this heterogeneity reflects the real-world clinical population of stroke survivors, it may have introduced confounding effects in the analysis of neurophysiological and functional outcomes. Third, no long-term follow-up was conducted in this study to assess the sustainability of the observed neuroplastic changes and functional improvements. Future studies with larger samples, stratified patient subgroups, and extended follow-up periods are encouraged to validate and extend the current findings. Fourth, no individual head measurements or 3D digitization were performed, which may have introduced minor spatial variability across participants. Fifth, channels overlying lesioned cortical tissue were not removed in the present study. Although some fNIRS studies have adopted lesion-based channel exclusion, there is currently no consensus on this practice. Future studies incorporating individualized lesion masking may help further refine connectivity estimates in stroke populations. Finally, the sham stimulation was delivered with a 90 deg coil tilt at the same site, which may not have fully matched the sensations of real rTMS and thus carries a risk of unblinding. Future studies should consider using an active sham coil or electrical scalp stimulation to achieve better sensory matching and improve blinding control.

## 6 Conclusion

This study demonstrated that low-frequency rTMS applied to the contralesional M1 promotes motor recovery in stroke patients by modulating both interhemispheric inhibition and functional connectivity, and rebalancing bilateral excitability. By combining paired-pulse TMS and fNIRS, we provide converging evidence that rTMS primarily reduces inhibitory drive from the contralesional to the ipsilesional hemisphere, potentially facilitating perilesional cortical reorganization. These findings offer new insight into the neural mechanisms underlying rTMS-induced recovery and highlight the value of integrating multimodal neurophysiological assessments. Future research should explore long-term effects, individual variability, and the impact of task-related modulation to optimize personalized neuromodulation strategies.

## Supplementary Material

10.1117/1.NPh.13.S1. S13010.s01

## Data Availability

The data that support the findings of this article are not publicly available due to privacy concerns. They can be requested from the author at dryangsiqi@gmail.com.
