# Supplementary material for: Investigating repetitive transcranial magnetic stimulation–induced interhemispheric changes in stroke: a transcranial magnetic stimulation and fNIRS study
Source: Neurophotonics. 2026 Feb 10;13(Suppl 1):S13010. doi: 10.1117/1.NPh.13.S1.S13010 (PMC12890182; doi:10.1117/1.NPh.13.S1.S13010)
Supplement: Supplementary file 1 [file NPh_013_S13010_SD001.pdf]

Table S1. fNIRS Channel Information

Channel	MNI			Brodmann area	Probability (%)
	X	Y	Z		
CH1	-14	20	68	BA6 - Pre-Motor and Supplementary Motor Cortex	44
CH2	-17	-5	75	BA6 - Pre-Motor and Supplementary Motor Cortex	100
CH3	-29	-5	70	BA6 - Pre-Motor and Supplementary Motor Cortex	100
CH4	-41	-4	63	BA6 - Pre-Motor and Supplementary Motor Cortex	90
CH5	-52	-4	55	BA6 - Pre-Motor and Supplementary Motor Cortex	80
CH6	-17	-27	78	BA4 - Primary Motor Cortex	87
CH7	-32	-27	74	BA4 - Primary Motor Cortex	75
CH8	-44	-27	68	BA4 - Primary Motor Cortex	43
CH9	17	17	69	BA6 - Pre-Motor and Supplementary Motor Cortex	63
CH10	19	-6	75	BA6 - Pre-Motor and Supplementary Motor Cortex	100
CH11	32	-7	70	BA6 - Pre-Motor and Supplementary Motor Cortex	100
CH12	43	-6	65	BA6 - Pre-Motor and Supplementary Motor Cortex	73
CH13	55	-6	54	BA6 - Pre-Motor and Supplementary Motor Cortex	65
CH14	21	-30	77	BA4 - Primary Motor Cortex	81
CH15	34	-29	74	BA4 - Primary Motor Cortex	72
CH16	45	-30	68	BA4 - Primary Motor Cortex	38

Table S2. MNI coordinates of the optodes

Channel	MNI		
	X	Y	Z
S1	-12	7	73
S2	-35	8	65
S3	-53	6	46
S4	-13	-39	80
S5	-38	-41	70
S6	20	28	62
S7	28	-17	75
S8	51	-18	63
D1	-16	32	60
D2	-25	-16	75
D3	-48	-16	61
D4	14	4	74
D5	38	7	64
D6	58	6	46
D7	15	-40	80
D8	38	-42	70

Table S3. Lesion characteristics of stroke participants.

ID	Group	Stroke type	Lesion side	Lesion location
1	TMS	Ischemic	Right	Basal ganglia
2	TMS	Ischemic	Left	Periventricular–basal ganglia
3	TMS	Ischemic	Left	Basal ganglia and periventricular area, frontoparietal cortex
4	TMS	Ischemic	Left	Brainstem, frontal lobe
5	TMS	Hemorrhagic	Right	Basal ganglia
6	TMS	Ischemic	Left	Basal ganglia, lateral ventricle, frontotemporal cortex
7	TMS	Ischemic	Right	Parietal cortex
8	TMS	Ischemic	Right	Frontotemporo-occipital-insular cortex
9	TMS	Ischemic	Right	Brainstem
10	TMS	Ischemic	Left	Basal ganglia region, periventricular area
11	TMS	Ischemic	Left	Pons
12	TMS	Ischemic	Left	Brainstem
13	TMS	Ischemic	Right	Pons
14	TMS	Ischemic	Left	Posterior limb of the internal capsule, temporal lobe
15	Sham	Hemorrhagic	Right	Thalamus, basal ganglia
16	Sham	Ischemic	Left	Brainstem
17	Sham	Ischemic	Left	Basal ganglia and periventricular area
18	Sham	Ischemic	Right	Basal ganglia and periventricular area
19	Sham	Ischemic	Right	Frontoparietal cortex, lateral ventricle
20	Sham	Ischemic	Right	Basal ganglia, centrum semiovale, frontotemporoparietal cortex
21	Sham	Ischemic	Right	Brainstem
22	Sham	Ischemic	Left	Cerebral hemisphere (middle cerebral artery territory)
23	Sham	Ischemic	Right	Basal ganglia, corona radiata
24	Sham	Ischemic	Left	Periventricular area, brainstem
25	Sham	Ischemic	Right	Pons
26	Sham	Hemorrhagic	Right	Periventricular–basal ganglia
27	Sham	Ischemic	Right	Cerebral hemisphere (middle cerebral artery territory)
